# Resistance to complement-mediated lysis of parainfluenza virus 5-infected cells is acquired after transition from acute to persistent infection

**DOI:** 10.1128/jvi.01895-24

**Published:** 2025-01-10

**Authors:** Candace R. Fox, Nasser N. Yousef, Namita Varudkar, Elisabeth M. Shiffer, Jenna R. Aquino, Kritika Kedarinath, Griffith D. Parks

**Affiliations:** 1 University of Central Florida, College of Medicine 124506 , Orlando, Florida, USA; University Medical Center Freiburg, Freiburg, Germany

**Keywords:** Complement, persistent infection

## Abstract

Persistent viral infections can be an important medical problem, with persistently infected (PI) cells extending viral shedding, maintaining inflammation, and providing potential sources for new viral variants. Given that PI cells can acquire resistance to some innate immune pathways, we tested the hypothesis that complement (C’)-mediated lysis of parainfluenza virus 5 (PIV5)-infected cells would differ between acute-infected and PI cells. Biochemical and real-time cell viability assays showed effective C’-mediated lysis of A549 lung cells acutely infected with PIV5, through pathways that depended on C3 and C5, but largely independent of C6. A PIV5 PI cell line established by long-term culturing of acutely infected A549 cells showed a high-level persistent expression of PIV5 proteins and infectious virus. Under conditions that led to effective lysis of acute PIV5-infected cells, the PI cells were nearly completely resistant to C’-mediated killing. This lack of C’ killing was not due to failure to activate C’, since C’-treated PIV5 PI cells had extensive C3 and membrane attack complex deposition, as well as production of C3a and C5a. Transcriptomics analysis revealed the C’ cascade as the most significantly upregulated pathway in PIV5 PI cells versus acute infection. Biochemical analyses showed that resistance to C’ killing correlated with increased expression in PI cells of two major C’ inhibitors: complement factor H and Vitronectin. The finding of acquisition of C’ resistance after the transition from acute PIV5 infection to PI cells raises the potential to inform therapeutics for PIs based on modulating C’ pathways.

**IMPORTANCE:**

A persistent infection (PI) with RNA viruses can extend virus shedding, prolong inflammation, and be a source of new viral variants. Since profound changes to innate immune pathways can occur in PI cells, it was important to test PI cells for changes in sensitivity to the complement (C') system, powerful innate immune pathways capable of lysing infected cells. Using parainfluenza virus 5 (PIV5) as a model system, we show that PI cells are nearly completely resistant to C'-mediated lysis, in stark contrast to high sensitivity of acute PIV5-infected cells to C' killing. A key finding was the upregulated expression in PI cells of two C' inhibitors: Vitronectin and complement factor H. These are important results with strong potential to inform therapeutics, given that polymorphisms in C' genes can correlate with severity of viral infections, and clinical trials are underway with new drugs that modulate C' responses.

## INTRODUCTION

RNA virus infections can transition from an initial acute infection to a prolonged or persistent infection (PI), in which the infected cells survive and continue to produce progeny virus (1, 2). This transition to PIs can extend the time period for production and shedding of infectious virus. In a changing persistently infected cell environment, continuous virus replication can also be a source of new viral variants that have altered abilities to spread through different tissues or escape neutralization (1, 3–6).

Some members of the paramyxovirus family of non-segmented negative strand RNA viruses are associated with PIs in human populations (7–10), as well as in animal reservoirs (11, 12). Measles virus, Sendai virus, Mumps virus, and parainfluenza viruses have also been shown to establish PIs in cell culture (5, 6, 13–19). Parainfluenza virus 5 (PIV5) readily establishes PIs in a large number of cultured cell lines, which can result in changes to virus replication including altered viral polymerase subunits (13), inclusion body formation (4, 20), and emergence of fusogenic mutants (5).

The acute-to-persistent transition for RNA virus infections can also lead to changes in innate immune pathways promoting evasion from host defense systems (15, 18, 21), thereby contributing to survival of the PI cells. In some cases, cells persistently infected with RNA viruses show altered interferon (IFN) signaling (18) or reduced sensitivity to cell death pathways (22). Complement (C’) is a powerful innate anti-viral system (23, 24), which, upon activation, leads to the recognition of microbes, direct neutralization of viruses, lysis of infected cells, and recruitment and activation of lymphocytes (25, 26). Although C’ can be a major contributing factor in inflammation during respiratory virus infections (27, 28), the role of C’ in controlling persistent paramyxovirus infections is not well understood. Here, we show dramatic differences in the outcome of C’ interactions with cultured human lung cells before and after the transition from an acute PIV5 infection to a PI.

The C’ cascade is initiated through three main pathways—classical, lectin, and alternative—converging on component C3, which is cleaved to C3a and C3b. This generates C5 convertase, and together with C6, C7, C8, and C9, it leads to formation of the membrane attack complex (MAC), which can lyse viruses or infected cells. To prevent inappropriate activation, soluble and membrane-bound cellular inhibitors act at various stages of the C’ cascade (23, 24, 29). For example, CD46, complement factor H (CFH), and CD55 can contribute to the inactivation of C3 and C5 convertases, whereas CD59, Clusterin, and Vitronectin (VTN) can inhibit MAC activity. Some cellular inhibitors can be exploited by viruses to evade C’-mediated neutralization and cell lysis (21, 30–32). For example, PIV5 recruits cellular C’ inhibitors CD55, CD46, and CD59 during the assembly budding process (33, 34). PIV5 infection upregulates cell surface CD55 to produce progeny virions containing increased levels of CD55 which leads to enhanced resistance to C’-mediated neutralization (35). However, the role of C’ inhibitors in persistent paramyxovirus infections and C’-mediated cell lysis is not well understood.

Here, we show that cultured human lung cells that are initially infected with PIV5 are highly sensitive to C’-mediated killing, through pathways that are dependent on C3 and C5 for cell lysis. Strikingly, after transition of the acute PIV5-infected cells to a long-term persistent PIV5 infection, the cells are nearly completely resistant to lysis by human C’ pathways. Consistent with this resistance to cell lysis, transcriptomics and biochemical data show that the PIV5 PI cells have increased expression of cellular C’ inhibitors CFH and VTN when compared to the initial acutely infected cells. Our work has implications for better understanding the complex interactions between host immune responses and chronic virus infections and may provide insight into more effective treatments.

## RESULTS

### NHS lyses acute PIV5-infected human lung cells and results in cell surface C3 and MAC deposition

**Fig 1:**
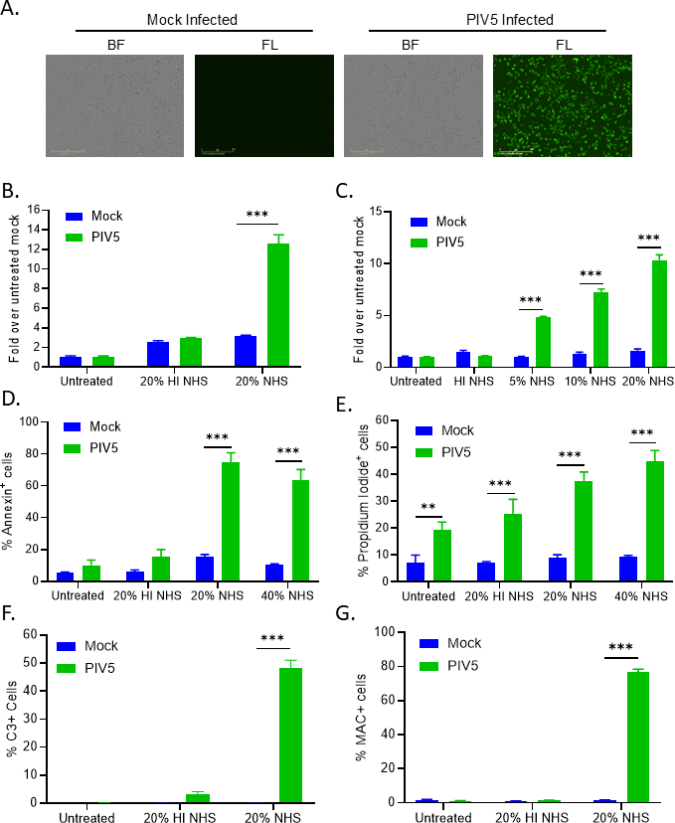
NHS treatment of PIV5-infected human lung cells results in cell lysis and deposition of C3 and MAC. (**A**) A549 cells were mock infected or infected with PIV5 at an MOI of 10 PFU/cell. At 18 hpi, cells were examined by bright field (BF) and fluorescence (FL) microscopy for GFP expression. (**B and C**) A549 cells were mock infected or infected with PIV5 at an MOI of 10. At 18 hpi, cells were either left untreated or treated with HI NHS or NHS and incubated for 4 h at 37°C, followed by CytoTox-Glo cytotoxicity assays. NHS concentrations were either 20% (panel B) or 5%, 10%, and 20% (panel C). (**D and E**) Alternatively, at 18 hpi mock infected or PIV5 infected cells were either left untreated or treated with 20% HI NHS or 20% and 40% NHS. Samples were analyzed by Annexin V (**D**) and propidium iodide (**E**) staining and flow cytometry. (**F and G**) A549 cells were mock infected or infected with PIV5 at an MOI of 10. At 18 hpi, cells were either left untreated or treated with 20% NHS, followed by antibody staining for C3 (**F**) or C5b-9 (**G**) and quantified by flow cytometry. Values in all panels are the mean of three replicates and error bars representing standard deviation. ** indicates *P*-value < 0.01 and *** indicates *P*-value < 0.001.

The Leader mutant PIV5 expressing GFP was generated previously by introducing two nucleotide substitutions, U5C and A14G, in the leader promoter region of recombinant PIV5-GFP virus (36). While similar results were seen with WT PIV5, this leader mutant (referred to here as PIV5) is used in these studies since it was shown previously to be a very strong activator of C’ pathways in acute infected human cells (31). To define the properties of C’ interaction with PIV5-infected cells, A549 human lung cells were either mock infected or infected with PIV5 at MOI of 10. At 18 hours postinfection (hpi), cells imaged under bright field (BF) or fluorescence (FL) showed the vast majority of PIV5-infected cells were expressing GFP and had minimal cytopathic effects (Fig. 1A). This timepoint after acute infection was chosen for the C’ analysis.

Normal Human Serum (NHS) was used as a source of C’ factors to determine the extent to which C’ lysed PIV5-infected cells. Mock infected or PIV5-infected cells were either untreated or treated for 4 h with 20% NHS. Samples treated with 20% heat inactivated (HI) NHS were included as a control for inactive C’. Cell death was then determined by a Cytotox-Glo assay. As shown in Fig. 1B, both mock and PIV5-infected cells showed minimal cytotoxicity when left untreated or treated with 20% HI NHS. Treatment with 20% NHS led to ~3 fold increase in cytotoxicity of mock infected cells compared to untreated mock, whereas PIV5-infected cells had a ~ 12 fold increase in cytotoxicity. As shown in Fig. 1C, NHS treatment resulted a dose-dependent killing of PIV5-infected cells. It is important to note that the commercially available human sera used in this study did not contain PIV5 cross-reactive antibodies capable of binding to PIV5-infected cells, as demonstrated in our previous work (31). This suggests differential mechanisms of C-mediate killing rather than antibody-dependent cytotoxicity.

As an alternative assay for cell viability, mock infected or PIV5-infected cells were either left untreated, treated with HI NHS as a control, or with 20% NHS or 40% NHS and incubated for 4 h. Cell viability was measured by Annexin V (Fig. 1D) or Propidium Iodide (Fig. 1E) staining. While mock infected cells had low basal level of Annexin V and Propidium Iodide staining, PIV5-infected cells left untreated or treated with control C’-inactivated NHS showed staining levels which were ~20%–25% of the population. Most importantly, treatment of PIV5-infected cells with NHS led to ~70% Annexin V positive and ~45% Propidium Iodide (PI) positive cell populations. Consistent with formation of a C’ end product MAC, the finding of increased cell permeability to PI indicates that NHS treatment of PIV5-infected cells resulted in cell lysis.

To determine levels of C3 deposition on PIV5-infected cells, mock infected and PIV5-infected cells were either left untreated or treated with NHS before staining cell surfaces with an antibody to C3 and analyzing by flow cytometry. As shown in Fig. 1F, ~50% of NHS-treated PIV5-infected cells stained positive for C3, compared to minimal staining on mock and untreated PIV5 infected cells. Similar results were seen with an antibody that recognizes the C5b-9 complex. As shown in Fig. 1G, ~75% of NHS-treated PIV5-infected cells stained positive for C5b-9 deposition. These data indicate that C3 and C5b-9 are deposited on the surface of PIV5-infected cells when treated with NHS.

We utilized our previously described real-time cell monitoring assay (31, 37) with an IncuCyte instrument to define the kinetics of C’-mediated killing of PIV5-infected lung cells. Using A549 cells that stably express a nuclear red fluorescent protein (A549-NLR), the IncuCyte instrument can record red fluorescence nuclei in real-time and at continuous intervals. A549-NLR cells were mock infected or infected with PIV5 at an MOI of 10. At 18 hpi, cells were incubated alone, with 10% HI NHS as a control for inactive C’ or with 10% NHS. At each timepoint, the number of red-labeled cells (red object counts, ROC) was determined per well, normalized to the ROC at time 0 (ROC^t0^) and expressed as a percentage of time zero when treatments were added (ROC/ ROC^t0^).

**Fig 2:**
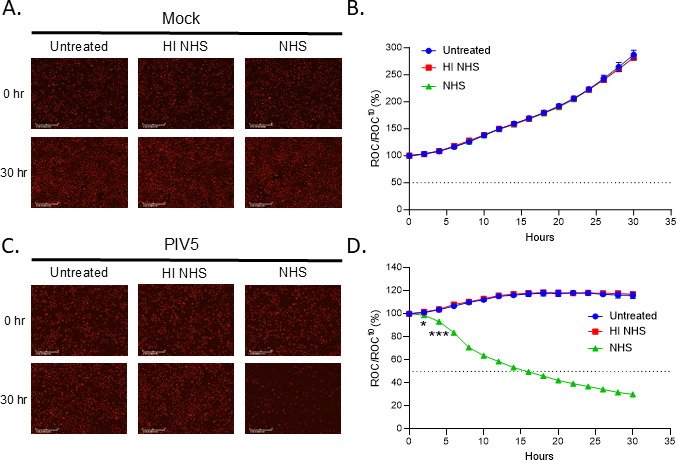
Real-time cell viability assays show NHS-mediated lysis PIV5-infected lung cells. A549-NLR cells expressing nuclear red fluorescent protein were mock infected (**A and B**), or infected with PIV5 (**C and D**) at an MOI of 10. At 18 hpi, cells were left untreated or treated with 10% HI NHS or 10% NHS. Bright field phase and red fluorescent images were recorded by real-time imaging using the IncuCyte instrument at 2 h intervals over 30 h. Representative images at 0 and 30 h are shown in panels (**A and C**). Red object count (ROC) per well was recorded by the IncuCyte and normalized to the value at time zero (ROC^t0^) when treatments were added and are expressed as percent of time zero (**B and D**). Values in all panels are the mean of three replicates and error bars representing standard deviation. * and *** indicate when a *P*-value of <0.05 and <0.001, respectively first appears in the time course comparing NHS-treated versus HI NHS-treated PIV5-infected samples, and this statistical significance is maintained at later timepoints.

Figure 2A and C show representative ROC images of mock infected and PIV5-infected cell at 0 time and at 30 h post-treatment with NHS or HI NHS. Figure 2B and D shows time courses of percentage of ROC normalized to time zero. As shown in Fig. 2B, mock infected cells that were untreated (blue curves) or treated with HI NHS or NHS (red and green curves, respectively) continued to proliferate to growth rates that ultimately reached ~300% of time zero. Figure 2D shows that untreated and HI NHS treated PIV5-infected cells (blue and red curves, respectively) increased to ~120% of time zero, consistent with prior reports that PIV5 infection results in a slower progression through the cell cycle (38). Most importantly, there was a significant loss of ROC compared to time 0 for the PIV5-infected cells that were treated with NHS (green curve), ultimately reaching ~50% of time zero by 15 h after NHS treatment.

### C’-mediated lysis of PIV5-infected cells requires C3 and C5, but is largely independent of C6

**Fig 3:**
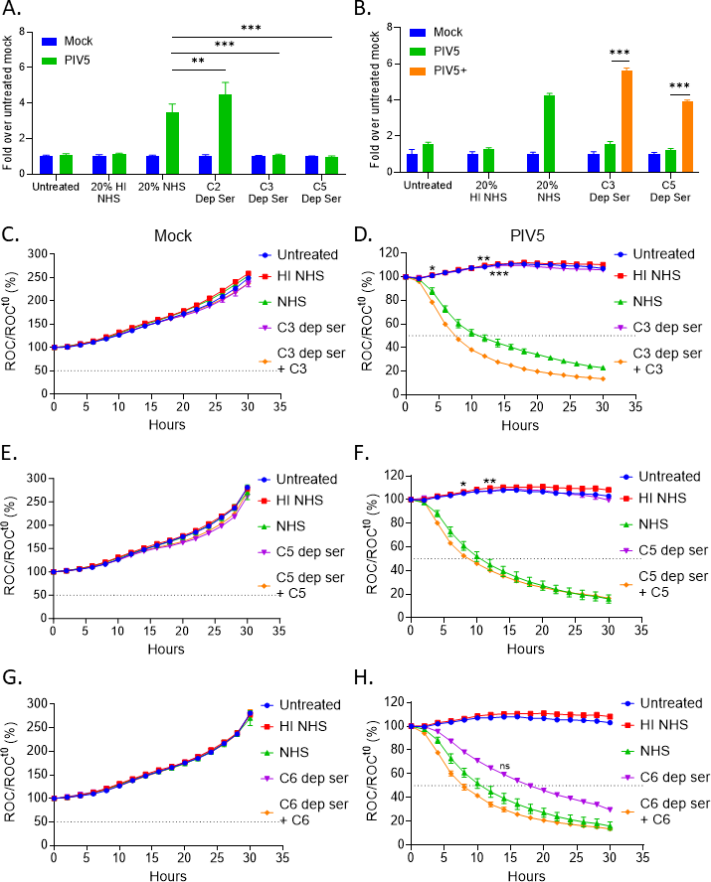
C3 and C5 are required for C’-mediated lysis of PIV5-infected lung cells. (**A**) A549 cells were mock infected or infected with PIV5 at an MOI of 10. Cells were left untreated or treated with 20% HI NHS, 20% NHS, or 20% C2-, C3-, or C5-depleted (dep) serum. Cells were incubated for 4 h at 37°C, followed by CytoTox-Glo cytotoxicity assays. (**B**) Mock infected or PIV5-infected A549 cells were untreated or were treated with 20% HI NHS, 20% NHS, 20% C3- or C5-dep serum, or 20% C3- or C5-dep serum supplemented with physiological levels of 100 µg/mL C3 and 75 µg/mL C5 (denoted PIV5+). Samples were analyzed by CytoTox-Glo cytotoxicity assays. (**C–H**) Red fluorescent images were acquired in real-time using the IncuCyte instrument at 2 h intervals over 30 h. At each time point, ROC per well was normalized to the value at time zero (ROC^t0^) when treatments were added and expressed as percent of time zero. For panels (**C and D**), 10% C3-dep serum or 10% C3-dep serum supplemented with physiological levels of C3 were tested as indicated. Similarly, 10% C5-dep serum alone or supplemented with C5 were tested as indicated in panels E and F. C6-dep serum (10%) alone or supplemented with C6 were tested as indicated in panels G and H. Values are the mean of three replicates with error bars representing standard deviation. *, **, and *** indicate when a *P*-value of <0.05, <0.01 and <0.001 respectively first appear in the time course, comparing PIV5-infected cells treated with NHS versus depleted sera (**D, F and H**). NS, not significant comparing C6 dep serum samples to NHS.

Our above data show deposition of the terminal MAC on PIV5-infected cells. To test for a functional role of components of the MAC in C’-mediated killing, mock infected or PIV5-infected A549-NLR cells were left untreated, or treated with 20% HI NHS, with NHS, or with sera individually depleted of C2, C3, or C5. After 4 h treatment, cytotoxicity was determined by a Cytotox-Glo assay and expressed as fold change compared to mock-infected untreated cells. As shown in Fig. 3A, PIV5-infected cells that were treated with C2-depleted sera had similar cytotoxicity levels compared to PIV5-infected cells treated with NHS. In contrast, treatment with C3- or C5-depleted sera resulted in basal levels of cytotoxicity. As shown in Fig. 3B, when physiological levels of C3 and C5 purified proteins were added back to C3- and C5-depleted sera (PIV5+, orange bars), lysis of PIV5 infected cells was restored. Although we did not validate the absence of C’ factors in depleted sera, the finding that a functional C’ pathway was restored when supplemented back with the missing purified C’ protein indicates that the function of the depleted sera depended on addition of the missing factor. These data indicate that NHS-mediated killing of PIV5 infected cells required C3 and C5 but not C2.

Real-time cell killing assays were carried out as described in the legend to Fig. 3 with C3-, C5-, or C6-depleted sera alone or when reconstituted with physiological levels of purified C3, C5, or C6. Cells were imaged at continuous intervals on the IncuCyte instrument, and ROCs were expressed as percentage of time zero. Figure 3C, E and G show that mock infected cells proliferated to ~250% of time zero under all treatment conditions. By contrast, PIV5-infected cells that were treated with C3- and C5-depleted sera (Fig. 3D and F, purple curves) showed curves similar to untreated and HI NHS-treated samples, consistent with the biochemical assays showing requirements for C3 and C5. Consistent with this, when purified C3 or C5 were supplemented back to their corresponding depleted sera (Fig. 3D and F, orange curves), the kinetics of loss of ROC for PIV5-infected cells showed a gradual decline similar to that seen with NHS-treated cells (green curves). Slightly different results were seen with C6-depleted serum (Fig. 3H), where loss of ROCs for PIV5-infected cells was slightly slower than that seen with NHS treated cells and this was increased by addition of purified C6. Taken together, these data suggest that PIV5-infected cells are targeted for C’-mediated cell lysis by a pathway that requires C3 and C5, with only a partially dependent of C6.

### PIV5 persistently infected cells activate C’ pathways but are resistant to C’-mediated lysis

**Fig 4:**
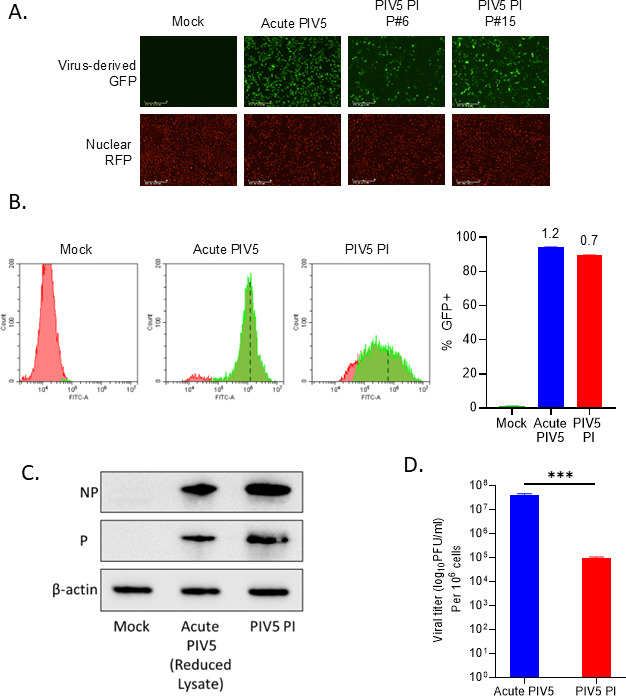
PIV5 persistently infected (PI) lung cells produce viral proteins and progeny virus. (**A**) A549-NLR cells were mock infected or infected with PIV5 at an MOI of 10 (acute PIV5 panel). Cells were passaged as a PI cell line and examined by fluorescence microscopy for GFP and RFP expression at passages (P) 6, and 15. (**B**) Mock, acute PIV5 infected, and PIV5 PI A549-NLR cells were analyzed by flow cytometry for GFP expression. Representative histograms of the FITC channel are shown, and the MFI was indicated by dashed lines. Percentage of GFP positive cells was determined, and the MFI is denoted above the bar graph. MFI are expressed in the magnitude of 1*10^6^. (**C**) Lysates from mock, acute PIV5 (24 hpi), and PIV5 PI cells were analyzed by western blotting for the indicated viral proteins. The β-actin blot shown here is the normalized gel with equal loading amounts for all samples. To optimize the viral protein western blots, lysates from the PI cells were loaded at three times the amount normalized to β-actin expression. The β-actin blots in Fig. 4C and 9E are duplicate images and the same lysates were used to probe for the corresponding proteins. (**D**) Virus released from PIV5 PI cells 24 h after cell plating or from an acute PIV5 infection at 24 hpi were quantified by plaque assay and normalized to 10^6^ cells. *** indicates *P*-value of <0.001.

WT PIV5 and PIV5 mutants are known to readily establish PIs of cultured cell lines (19). We established an A549 PI cell line by high MOI acute infection, followed by growth and passage of infected cells. As shown in Fig. 4A, the majority of PIV5 PI cells continued to express GFP at passage (p) numbers 6 and 15. By flow cytometry analysis, approximately 90% of the PIV5 PI cell population was positive for GFP expression and had a mean fluorescence intensity (MFI) of 0.7 × 10^6^ compared to an MFI of 1.2 × 10^6^ for acute infected cells (Fig. 4B). As determined by western blotting for NP and P expression (Fig. 4C), these PI cells produced viral proteins at ~3–4 fold lower levels than seen in a typical acute infection. In addition, these PIV5 PI cells continued to produce infectious virus with titers approximately 2.5 logs lower than virus production from the acute PIV5-infected cells as quantified by plaque assays (Fig. 4D). These results demonstrated the establishment of an A549 PIV5 PI cell line for our C’ studies.

**Fig 5:**
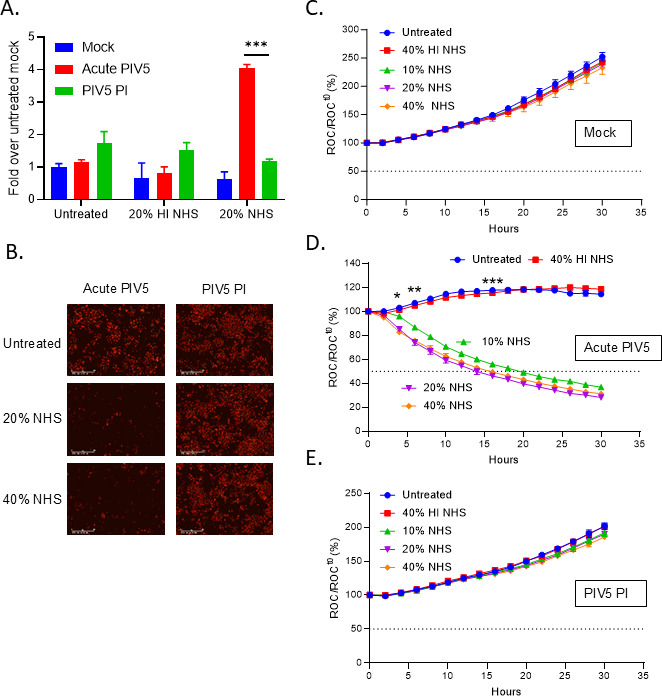
PIV5 PI cells are resistant to C’-mediated lysis. (**A**) A549 cells that were mock infected, PIV5 infected, or PIV5 PI A549 cells were left untreated or treated with 20% HI NHS, or 20% NHS. Cells were then incubated for 4 h at 37°C, followed by CytoTox-Glo cytotoxicity assays. (**B–E**) Mock infected, acute PIV5 infected or PIV5 PI A549-NLR cells were untreated or treated with 40% HI NHS, or either 10%, 20%, or 40% NHS. Red fluorescent images were recorded by real-time imaging using the IncuCyte instrument at 2 h intervals over 30 h. Representative images at 30 h post treatment are shown in panel B. ROC per well was calculated and normalized to the value at time zero (ROC^t0^) when treatments were added and expressed as percent of time zero (**C–E**). Values are the mean of three replicates with error bars representing standard deviation. *, **, and *** indicate when a *P*-value of <0.05, <0.01, and <0.001 respectively first appear in the time course comparing NHS treated versus HI NHS treated PIV5-infected samples.

To determine the outcome of C’ interactions with the PIV5 PI cells, mock infected, acute PIV5-infected, and PIV5 PI cells were either left untreated or treated for 4 h with 20% HI NHS or 20% NHS. Cell death was then determined by the Cytotox-Glo assay and expressed as fold over untreated mock. Consistent with our above data, NHS-treated acute PIV5-infected cells showed about a 4-fold increase in cytotoxicity (red bars, Fig. 5A). Remarkably, NHS-treated PIV5 PI cells had similar cytotoxicity levels as NHS-treated mock cells (compare green and blue bars, Fig. 5A) indicating a lack of C’-mediated cell lysis.

As an alternative sensitive assay, NHS dose-response experiments were conducted with real-time cell viability assays on the IncuCyte (Fig. 5B through E). Mock infected, acute PIV5-infected, and PIV5 PI cells were either left untreated or treated with 10%, 20%, or 40% NHS or 40% HI NHS as a control. Representative images shown in Fig. 5B demonstrate a clear difference between the loss of cells seen in the NHS-treated acute PIV5-infected wells compared to the proliferation observed in the NHS-treated PIV5 PI cells. In real-time ROC assays, mock-infected cells proliferated to ~250% of time zero under all treatment conditions (Fig. 5C). Acute PIV5-infected cells treated with 10%, 20%, and 40% NHS showed a time-dependent decline in cell viability across the various doses (Fig. 5D). Most strikingly, PIV5 PI cells treated with increasing NHS doses continued to proliferate to ~200% of time zero, with very similar ROC increases as the untreated PIV5 PI cells (Fig. 5E). Taken together, these biochemical and real-time viability data show that PIV5 PI cells were resistant to C’-mediated lysis.

**Fig 6:**
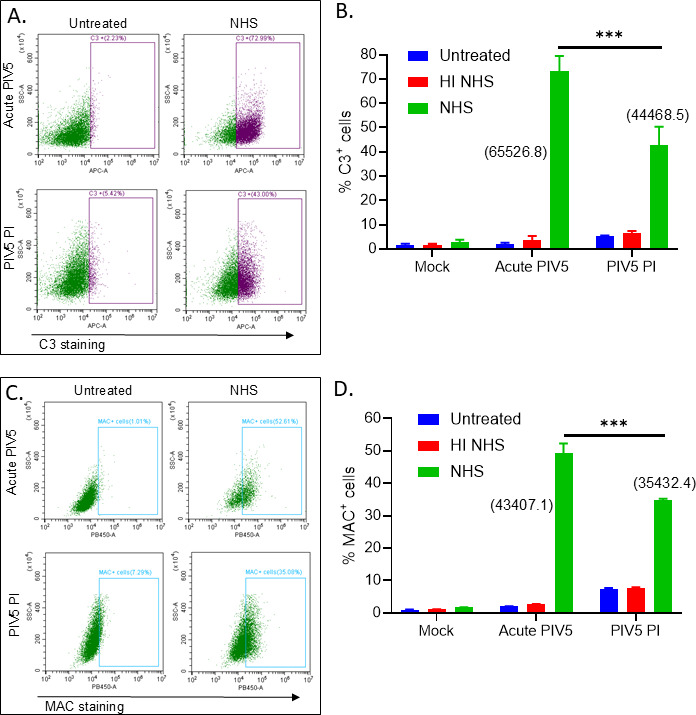
NHS treatment of PIV5 PI cells results in surface deposition of C3 and MAC. (**A–D**) Mock infected, acute PIV5-infected, or PIV5 PI A549 cells were either left untreated or treated with 20% HI or 20% NHS. Reactions were incubated for 2 h, followed by antibody staining for C3 (**A and B**) and C5b-9 (**C and D**) deposition and quantified by flow cytometry. Representative scatterplots are shown in panels (**A and C**). Values in all panels are the mean of three replicates with error bars representing standard deviation. *** indicating *P*-value of <0.001.

The above resistance to C’ lysis could be explained by either: (1) PIV5 PI cells fail to activate C’ pathways or (2) C’ is activated, but PIV5 PI cells are able to inhibit C’-mediated cell lysis. To test for C’ activation, mock infected, acute PIV5-infected, and PIV5 PI cells were treated with NHS and then antibody stained for surface deposition of C3 and C5b-9 (Fig. 6C and D). As shown in Fig. 6A and B, C3 staining was seen in ~40% of NHS-treated PIV5 PI cells with an MFI of 44468. This was significantly lower than 75% of cells staining and MFI of 65527 for acute infected cells. Similarly, PI and acute infected cells showed ~35% and ~48% staining for MAC components, respectively, with MFIs of 35432 and 43407 (Fig. 6C and D). Importantly, these results show that, despite being lower than NHS-treated acute infected cells, the C3 and C5b-9 surface staining was still very high on the NHS-treated PI cells. Thus, the lack of C’-mediated lysis of PI cells cannot be explained solely by lack of C3 or MAC deposition.

### Unbiased global transcriptomics analysis reveals the C’ cascade is the most significantly altered pathway in the PIV5 PI cells

**Fig 7:**
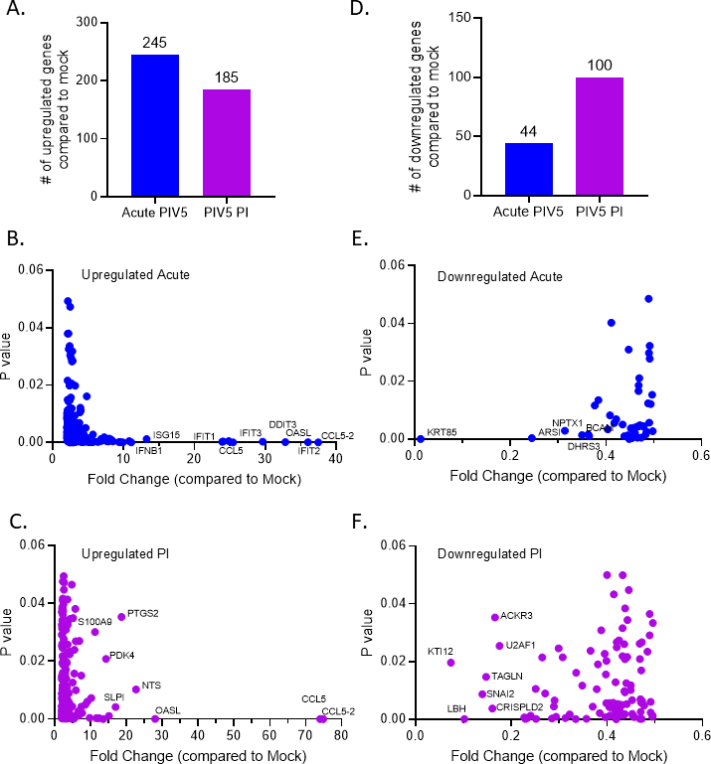
Global transcriptomic analysis of gene expression in acute PIV5 infection and PIV5 PI cells. (**A–F**) A549 cells were mock infected or infected with PIV5 at an MOI of 10, or PIV5 PI cells were plated. At 18 hpi, RNA was extracted and sequenced to identify statistically significant differentially expressed genes comparing mock infected to either PIV5 acute infected cells or to PIV5 PI cells. The number of statistically significant genes that were upregulated (**A**) or downregulated (**D**) in the acute PIV5-infected cells or in the PIV5 PI cells compared to mock samples are shown. Scatterplots compared the fold change of the statistically significant genes that were either upregulated (**B**) or downregulated (**E**) in the acute PIV5-infected cells as compared to mock. Scatterplots compared the fold change of the statistically significant genes that were either upregulated (**C**) or downregulated (**F**) in the PIV5 persistently infected cells as compared to mock.

To determine the global changes to the landscape of gene expression during an acute PIV5 infection versus a persistent infection, unbiased RNA sequencing was performed. RNA was extracted from A549 cells that were either mock infected, acute PIV5-infected, or from PIV5 PI cells. RNA was then sequenced and analyzed for statistically significant differentially expressed genes. Acute PIV5-infected A549 cells showed 245 upregulated genes compared to mock controls, whereas PIV5 PI cells had 185 upregulated genes over mock (Fig. 7A). Acute PIV5-infected cells showed 44 downregulated genes compared to mock, whereas PIV5 PI cells had 100 downregulated genes over mock (Fig. 7D).

Statistically significant differentially expressed genes in the acute PIV5 infection versus mock infection were analyzed on scatterplots to better understand the host response to an acute PIV5 infection. Highly upregulated genes in the acute PIV5-infected cells mainly involved interferon stimulated genes, such as *CCL5-2, IFIT2, OASL, DDIT3, IFIT3, CCL5, IFIT1, ISG15,* and *IFNB1* (Fig, 7B), while highly downregulated genes were *KRT85, ARSI, NPTX1, DHRS3,* and *BCAM* (Fig. 7E). These global transcriptomics studies highlighted the hundreds of genes that are activated in response to acute PIV5 infection. In contrast, highly upregulated genes in the PIV5 PI samples compared to mock included *CCL5-2, CCL5, OASL, NTS, PTGS2, SLPI, PDK4,* and *S100A9* (Fig. 7C)*,* whereas highly downregulated genes included *KTI12, LBH, SNAI2, TAGLN, CRISPLD2, ACKR3,* and *U2AF1* (Fig. 7F).

**Fig 8:**
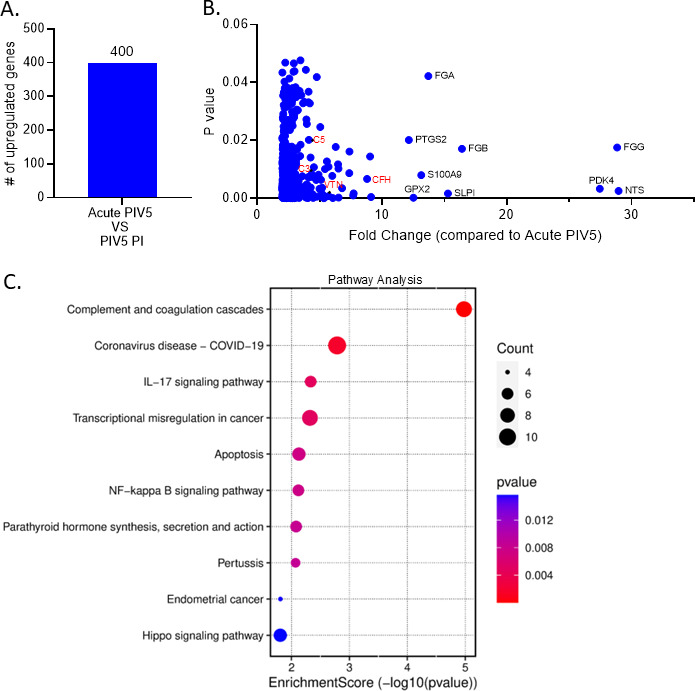
The complement pathway is the most significantly altered pathway in PIV5 PI cells compared to acute PIV5 infection. RNA samples prepared as described in legend to Fig. 7 were further analyzed to determine the number of statistically significant genes upregulated in the PIV5 PI cells compared to acute PIV5-infected cells (panel A). The fold change of statistically significant upregulated genes in the PIV5 PI cells compared to acute PIV5-infected cells are represented in a scatterplot in panel B. Panel C shows a gene ontology (GO) pathway enrichment dot plot with the circle size corresponding to the number of genes present in the associated pathway and the color scale corresponding to the *P*-value. The gene ratio is represented on the horizontal axis and the enriched pathway is listed on the vertical axis.

As a key comparison, the number of upregulated genes in the PIV5 PI samples was compared to the acute PIV5-infected samples. Four hundred genes were specifically upregulated in the PIV5 PI cells compared to acute PIV5-infected cells (Fig. 8A), including *NTS, PDK4, FGG, C3, C5, VTN,* and *CFH* (Fig. 8B). GO pathway enrichment analysis identified the most significantly altered pathways in the PIV5 PI cells compared to the acute PIV5-infected cells (Fig. 8C). Remarkably, the most significantly differentially altered pathway was the C’ cascade.

**Fig 9:**
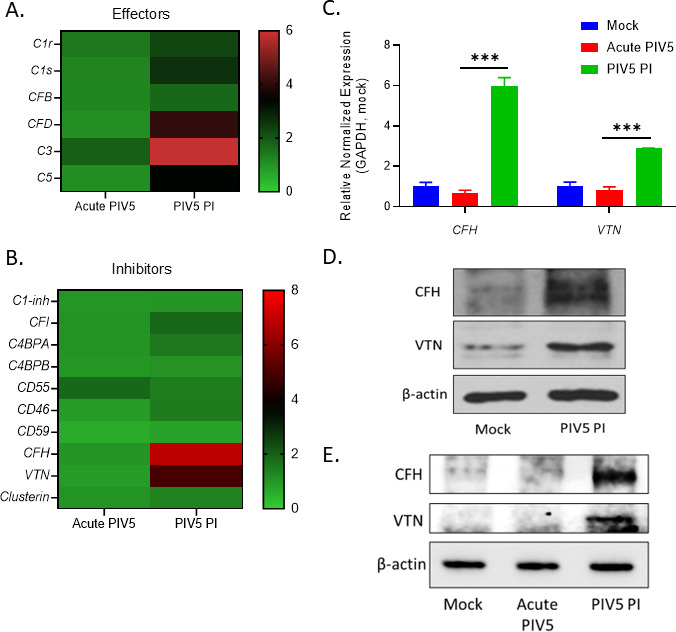
Expression of complement inhibitors VTN and CFH are upregulated in the PIV5 PI cells. (**A and B**) Using mock infected samples as a baseline, RNA samples prepared as described in the legend in Fig. 7 were further analyzed to generate heatmaps to visualize fold changes in gene expression of complement effectors (**A**) or inhibitors (**B**) that were either upregulated (red, values > 1) or downregulated (green, values < 1) in acute PIV5-infected cells or the PIV5 PI cells. (**C**) RNA samples collected as described in legend to Fig. 7 were analyzed by RT-qPCR to determine gene expression of *CFH* and *VTN*. (**D and E**) Protein lysates collected at 18 hpi from mock infected, acute PIV5 infected, or PIV5 PI cells (18 hp plating) were analyzed by western blotting for levels of actin, VTN, and CFH proteins. The β-actin blots in Fig. 4C and Fig. 9E are duplicate images and the same lysates were used to probe for the corresponding proteins. Western blot band intensities were measured using ImageJ software and the relative band quantification ratios were calculated and normalized to mock lysate. The relative band quantification ratios of CFH were 13- and 108-fold increase in the respective acute and PI lysates, as compared to the mock CFH band intensity. The relative band quantification ratios of VTN were 1.4- and 3.6-fold increase in the respective acute and PI lysates, as compared to the mock VTN band intensity.

Heatmaps of C’ pathway effector genes (Fig. 9A) and C’ inhibitor genes (Fig. 9B) were generated comparing either the acute PIV5-infected cells or the PIV5 PI cells to mock as a baseline. Most strikingly, both C’ effector genes *C3, C5,* and *CFD* as well as C’ inhibitor genes *VTN* and *CFH* were upregulated in the PIV5 PI cells. Using qPCR on RNA from mock infected, acute and PI cells, differential levels of CFH and VTN RNA were found to be very similar to the fold changes predicted in heat maps (Fig. 9C). Western blotting confirmed higher levels of VTN and CFH protein in lysates from PIV5 PI cells compared to mock infected cells (Fig. 9D). This was further confirmed comparing levels of CFH and VTN protein in lysates from acute infected versus PI cells (Fig. 9E). Compared to the mock CFH band intensity, the relative band quantification ratios of CFH were 13- and 108-fold increase in acute and PI lysates, respectively. The relative band quantification ratios of VTN were 1.4- and 3.6-fold increase in the respective acute and PI lysates, as compared to the mock VTN band intensity. Taken together, these data show a strong correlation between differential sensitivity and resistance of acute PIV5 infected cells versus PI cells to C’-mediated lysis and the differential increased expression of C’ inhibitors including CFH and VTN.

### Multiple factors contribute to C’-mediated lysis resistance in PIV5 PI cells

**Fig 10:**
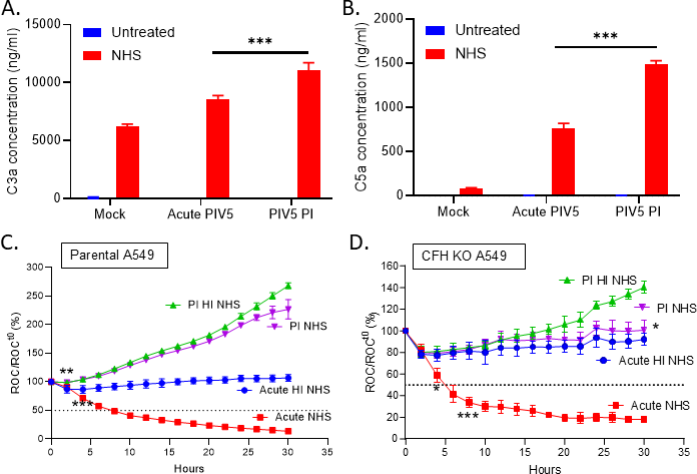
PIV5 persistently infected CFH knock out A549 cells are resistant to C’-mediated lysis. (**A and B**) Mock infected, acute PIV5-infected, or PIV5 PI A549 cells were left untreated or treated with NHS for 4 h. Supernatants were collected and analyzed for C3a (panel A) and C5a (panel B) by ELISA and normalized to 10^6^ cells. (**C and D**) Acute PIV5 infected or PIV5 PI A549-NLR cells (panel C) or CFH Knock-Out (KO) A549-NLR cells (panel D) were untreated or treated with either 10% HI NHS or 10% NHS. Red fluorescent images were recorded by real-time imaging using the IncuCyte instrument at 2 h intervals over 30 h. ROC per well was calculated and normalized to the value at time zero (ROC^t0^) when treatments were added and expressed as percent of time zero. Values are the mean of three replicates with error bars representing standard deviation. *, **, and *** indicate when a *P*-value of <0.05, <0.01 and <0.001 respectively first appear in the time course comparing NHS treated PIV5 PI cells versus NHS treated PIV5-infected samples.

Since the C’ inhibitor CFH was upregulated in the PI cells and CFH can contribute to inactivation of C3 and C5 convertases leading to overall C’ activation reduction, levels of C3 and C5 activation were determined. C3a and C5a levels were measured by ELISA in media from NHS-treated mock infected, acute infected, and PIV5 PI cells as an indication of C’ activation. As shown in Fig. 10A and B, both C3a and C5a were found in higher levels in the case of NHS-treated PIV5 PI cells compared to acute PIV5-infected cells. These data indicate under conditions that lead to lysis of acute PIV5 infected cells, PI cells are resistant to C’-mediated killing despite having higher activation of C’ to produce C3a and C5a.

To determine the functional role of CFH in C’-mediated lysis resistance in the PIV5 PI cells, commercially available human CFH knockout (CFH KO) A549 cells were engineered to express nuclear red fluorescent protein and a PIV5 PI cell line was created as described above. NHS treatment experiments were then conducted with real-time cell viability assays on the IncuCyte instrument. Acute PIV5-infected or PIV5 PI parental A549-NLR cells (Fig. 10C) and CFH KO A549-NLR cells (Fig. 10D) were treated with either 10% NHS or 10% HI NHS as a control. As shown in the kinetic analysis in Fig. 10C and D, both parental and CFH KO acute PIV5-infected cells treated with 10% NHS showed a very similar time-dependent decline in cell viability (red curves). Consistent with the above results, PIV5 PI parental A549 cells treated with NHS continued to proliferate to ~250% of time zero (purple curves), with very similar ROC increases as the HI NHS treated control PIV5 PI cells (Fig. 10C). Most interestingly, PIV5 PI CFH KO A549 cells treated with NHS (purple curve), showed lower levels of ROC cells compared to the control PI cells treated with the C’-deficient HI NHS (green curve, Fig. 10D). Taken together, these real-time viability data show that knock out of CFH in A549 cells is not sufficient by itself to completely convert the PI cells from being resistant to C’-mediated lysis to the same level of sensitivity seen with acute infected cells. Thus, CFH is not the sole factor responsible for PI cells being resistant to C’-mediated lysis and that multiple factors contribute to this resistance.

## DISCUSSION

Persistent or prolonged viral infections can be an important clinical problem, with PI cells initiating and maintaining inflammation cascades (28) and providing potential sources for emerging new viral variants (1, 3, 5). In COVID patients, for example, C’ is a major pathway that is upregulated during early infection and often sustained during long COVID (39). Viruses have mechanisms to block C’-mediated cell lysis, such that a PI can set up a prolonged inflammation cycle - activated C’ continues to provide damaging inflammation, but viral inhibitory mechanisms block elimination of pathogen and infected cells. Given that PI cells can acquire resistance to innate immune pathways (15, 18, 21), we have tested the hypothesis that C’-mediated lysis of PIV5 infected cells would differ between acute infected and PI cells.

Prior work has shown that the removal of sialic acid by the PIV5 neuraminidase can activate the alternative C’ pathway (40). Consistent with this, we show that acute PIV5 infected cells are recognized and lysed through C’ pathways that depend on C3 and C5, but not C2 which is part of the classical/lectin pathways. Our finding that sera depleted of the MAC component C6 are still able to lyse acute PIV5 infected cells suggests that for some virally infected cells, all of the MAC components C5-C9 are not absolutely required for effective cell lysis at least in cell culture systems. This finding with PIV5 is also consistent with our prior work on C’ lysis of cells infected with the coronavirus OC43 (31), where cell lysis required the MAC components C5 and C6 but not C7. The molecular basis for cell type- and virus-specific requirements for the MAC subunits to function in lysis of infected cells will be an area of important future work.

Our most striking result was seen from our testing of the sensitivity of PI cells to C’-mediated lysis and the finding of nearly complete resistance. This resistance to lysis was not simply due to failure of the PI cells to activate C’, since we showed that C3 and MAC were deposited on PI cells, albeit at slightly lower levels compared to acute PIV5 infection. Likewise, despite resistance to C’ lysis, NHS-treated PI cells produced large amounts of C3a and C5a, indicating strong activation of the C’ cascade. Together, these data support the conclusion that resistance to C’ lysis of PI cells is due to active inhibition of C’ pathways, rather than differences in C’ activation.

Unbiased global transcriptomics analysis showed that significant gene expression patterns changed after the transition from an acute PIV5 infection to a PI. Four hundred genes were significantly upregulated in the PIV5 PI cells compared to acute PIV5-infected cells (Fig. 8A), including *NTS, PDK4, FGG, C3, C5, VTN,* and *CFH* (Fig. 8B). Remarkably, the C’ cascade was the most significantly altered pathway in the PIV5 PI cells compared to the acute PIV5-infected cells (Fig. 8C). Among these genes, C3 and C5 were significantly upregulated at the RNA level, indicating synthesis of these two effector proteins in the PI cells. Liver and serum are major sources of steady-state C’ levels in tissues (23, 24). However, it has been shown that C’ factors can also be synthesized locally by either infected cells or recruited lymphocytes (41–43). Recent work shows that C’ effectors (e.g., C3) can be expressed by non-hepatic and non-lymphoid cells (32, 43–46), and can act intracellularly as part of a “complosome” (46). Roles of intracellular C’ proteins and their importance in regulating or responding to viral infections remain unclear and are a very active area of interest. As such, our future work will determine the consequences of intracellular C’ proteins in the context of establishing a PI and regulating PI virus production, as well as determining their roles in resistance to C’-mediated lysis.

Consistent with the acquired resistance of PIV5 PI cells to C’-mediated lysis, our transcriptomics and biochemical analyses (RT-qPCR and western blotting) showed PI cells having increased expression at the RNA and protein levels of two major C’ inhibitors: Complement Factor H (CFH) and Vitronectin (VTN). Selective upregulation of these two C’ inhibitors (versus other inhibitors such as CD55, CD59, or CD46) raises important questions about what cellular signaling pathways drive expression from *VTN,* and *CFH* promoters. Similarly, these data raise the question of what viral factors or replication products contribute to this selective increased expression of C’ inhibitors. It is important to note that it is likely that resistance of PI cells to C’ lysis also includes contributions from other C’ inhibitors, such as CD55 and CD46 which we have shown to be key factors in resistance of PIV5 virions to C’-mediated neutralization (34). This redundancy in landscape of C’ inhibitors may reflect contributions through basal levels of expression as well as the high induction seen in the case of VTN and CFH. Consistent with this, our findings with CFH KO cells (Fig. 10C and D) show that deletion of this single factor is not sufficient by itself to completely convert PI C’-resistance to the C’-sensitivity seen with acute infected cells. Our results suggested that there is sufficient redundancy in C’ inhibitors to prevent identification of single factors that can completely convert the PI to acute C’ sensitivity.

Interestingly, the PIV5 PI cells are consistently maintained in culture by growth in media containing heat inactive serum (HI). Thus, in the absence of functional C’ pathways that can lyse cells, it is not clear what pressures exist on PI cells to increase substantial expression of just these two C’ inhibitors. One possibility is that the expression of effectors C3 and C5 in PI cells (Fig. 9A) needs to be countered by expression of C’ inhibitors such CFH and VTN in order to maintain cell viability. Alternatively, CFH and VTN may play non-canonical roles in cell growth outside of C’ inhibition. For example, intracellular CFH in some cancer cell types has been shown to influence cell migration and proliferation (47). An interesting observation supporting this hypothesis is the HI NHS treated control PIV5 PI CFH KO A549 cells did not proliferate to the same levels as the parental PIV5 PI A549 cells (Fig. 10C and D). VTN is known to play important roles in cell adhesion, migration, and spread (48). Consistent with these alternative functions for VTN outside of canonical C’ inhibition, our attempts to generate stable cell lines using CRISPR knockout of VTN expression in mock and PIV5 PI cells have not been successful.

PI cells can have an altered antiviral state induced by low levels of IFN and the production of IFN stimulated genes as seen previously with Newcastle Disease Virus infection (49, 50). IFN signaling could play a role in the lower virus production from PIV5 PI cells compared to an acute infection (Fig. 4D), but could also drive accumulation of mutations in the virus population. Consistent with this, measles virus PIs which lead to subacute sclerosing panencephalitis infections have mutations in the M, H, and F genes due to hypermutation events and polymerase errors (51). Since virus released from the PIV5 PI cells was less than from the acute PIV5 infection (Fig. 4D), we hypothesize the PI virus has acquired mutations that impair virus production and enhance persistence. An interesting question is whether the PI cell-derived progeny virus is also resistant to C’-mediated neutralization.

Results from our work on interactions of C’ with persistent RNA virus infections have strong potential to inform therapeutics, given: (i) the clinical impact of prolonged viral respiratory infections, (ii) polymorphisms in C’ genes can correlate with severity of viral infections (52, 53), and (iii) clinical trials are underway with new drugs that modulate C’ responses (54).

## MATERIALS AND METHODS

### Cells, viruses, and serum

Cultures of A549, Vero, and CV-1 cells were grown in Dulbecco modified Eagle medium (DMEM) supplemented with 10% heat inactivated fetal calf serum (HI FBS, Gibco, Thermo Fisher Scientific, Waltham, MA). Nuc-Light Red A549 cells expressing a nuclear red fluorescent protein (A549-NLR cells) were purchased from Sartorius (Sartorius, Göttingen, Germany). Human Complement Factor H (CFH) knockout A549 cells were purchased from Abcam (Cambridge, England) and were engineered to express a nuclear red fluorescent protein using the Incucyte Nuclight Red Lentivirus (puro) purchased from Sartorius (Sartorius, Göttingen, Germany).

Viruses were grown in Vero cells and titered on CV-1 cells as described previously (36). The Leader mutant PIV5 expressing GFP was generated previously (36). To obtain PI cell lines, A549 cells were infected with PIV5 at a multiplicity of infection (MOI) of 10 PFU/cell, and media was replaced every 3 days post infection (pi) for 2 weeks. After that PI cells were passaged and maintained as done for naïve A549 cells.

Pooled normal human serum (NHS) was purchased from Innovative Research (Novi, MI). Sera were heat inactivated (HI) at 56°C for 30 min. Sera individually depleted of C2, C3, or C5, and their purified proteins were purchased from Complement Technologies (Tyler, TX). Depleted sera were validated for functional loss of the specific factor by the manufacturer. Purified concentrated C3 (catalog number A113c) and C5 (catalog number A120) proteins were purchased from Complement Technologies.

### Cell viability assays

Cell viability assays were performed using the IncuCyte SX5 Live-Cell Analysis system (Sartorius) as previously described (31, 37). Briefly, A549-NLR cells were plated in triplicate in 96-well plates at 7,000 cells/well, then mock infected or infected with PIV5 at an MOI of 10 and maintained for 18 hours (h) before various treatments as indicated in figure legends. Plates were maintained in the IncuCyte CO2 incubator with microscopic images captured every 2 h using a 10× objective in red, green, and phase channels. Red object count (ROC) corresponding to cellular nuclei was calculated for each well. The numbers of ROC at each timepoint was normalized to the value at time zero (ROC_t0_) when treatments were added.

Biochemical cytotoxicity assays were performed using CytoTox-Glo assay (Promega, Madison, WI). A549 cells were plated in white-walled 96-well plates and infected as described above. At 18 hpi, NHS was added and incubated for 4  h at 37°C. CytoTox-Glo reagent was added to cultures and assayed according to manufacturer’s guidelines.

### Flow cytometry

For flow cytometric viability assays, cells were treated as indicated in each figure legend. Media and trypsinized adherent cells were collected for annexin V binding (BD Bioscience, Franklin Lakes, NJ) and propidium iodide (BD Bioscience) staining as described by the manufacturer. Cells were analyzed by flow cytometry using the CytoFLEX (Beckman Coulter, Brea, CA) and 10,000 independent events were recorded and analyzed using CytExpert software.

For C3 and C5b-9 staining, 48-well dishes of cells were treated as described in each figure legend, and cells were washed and harvested. Cells were then surface stained with C3 antibody (204869, Calbiochem, San Diego, CA), or SC5b-9 antibody (A239, Quidel, San Diego, CA), followed by appropriate secondary antibody staining using AlexaFluor633 antibodies (Invitrogen, Thermo Fisher Scientific). Cells were analyzed by flow cytometry as described above.

### Human C3a and C5a ELISAs

Six-well dishes of cells were treated as detailed in each figure legend, and media samples were evaluated using human C3a and C5a ELISA kits as described by the manufacturer (BD Opt EIA; BD Biosciences). ELISA results were normalized to 10^6^ cells.

### Western blotting

Six cm diameter dishes of cells were treated as described in the figure legends, followed by lysis in protein lysis buffer (Cell Signaling Technology Danvers, MA). Cell lysate was resolved on 12% SDS-PAGE gels (Bio-Rad, Hercules, CA) and transferred to nitrocellulose membranes. Samples were normalized to levels of β-actin (1:10,000 dilution, A5316, Sigma-Aldrich, St. Louis, MO) and equivalent levels of protein were analyzed with antibodies for VTN (1:1000 dilution, GTX103475, GeneTex, Irvine, CA), Complement Factor H (1:1000 dilution, A237, Complement Technologies), or rabbit polyclonal antisera to the PIV5 NP and *P* proteins (55). Blots were visualized by horseradish peroxidase-conjugated antibodies (Sigma-Aldrich) and chemiluminescence (Thermo Fisher Scientific). The β-actin blots in Fig. 4C and Fig. 9E are duplicate images and the same lysates were used to probe for the corresponding proteins shown in Fig. 4C and Fig. 9E. Western blot band intensities were measured using ImageJ software (https://ij.imjoy.io/) and relative band quantification ratios were calculated as detailed https://www.yorku.ca/yisheng/Internal/Protocols/ImageJ.pdf.

### Global transcriptomics

Cells were seeded in six cm diameter dishes and treated as indicated in figure legends. At 18 hpi, total RNA was extracted using the RNeasy Plus Mini Kit (QIAGEN, Germantown, MD) as per manufacturer’s instructions and subsequent quality control was performed using Nanodrop (Thermo Scientific) to ensure A260/280 ratio of ~2.0. RNA samples were then diluted to 50 ng/mL and shipped to Genewiz (South Plainfield, NJ) for RNA-sequencing. RNA (1.5 µg) was used to select polyadenylated RNA sequencing libraries with the Illumina RNA prep kit. RNA integrity, library size, and concentrations were evaluated on the Agilent TapeStation and with the Qubit instrument (LifeTech) according to GeneWiz specifications. Libraries were then sequenced on an Illumina HiSeq 2500 2 × 150 bp with a single index and paired-end reads were generated.

Raw FASTQ files were processed using the “new Tuxedo” pipeline (56). Briefly, reads that contained adapter sequences and low quality reads were removed using Trimmomatic. Subsequent clean reads were further analyzed using FastQC to ensure only high quality reads are processed. Clean reads were then aligned to the human genome (version GRCH38) using HISAT2. Stringtie and Ballgown in R were then utilized to identify differentially expressed genes and statistical analyses were performed.

Fold changes of genes and *P*-values were visually represented via scatterplots and heat maps generated in GraphPad Prism software with * indicated a *P*-value less than 0.05. A GO pathway enrichment dot plot was generated comparing acute PIV5-infected cells to PIV5 PI cells by analyzing statistically significant genes with fold changes greater than 2.5 using clusterProfiler, pathview, and ggplot2 in R and SRplot (57–60).

### Reverse transcription and quantitative real-time PCR (RT-qPCR)

Six-well dishes were treated as indicated in the figure legends, and RNA extraction was performed using TRIzol (Invitrogen) as described previously (19). TaqMan Reverse Transcription Reagents (Applied Biosystems, Foster City, CA) were used to obtain cDNA from 1 µg of total RNA (as per the manufacturer’s instructions). Bio-Rad CFX Connect Real-Time and Fast SYBR FAST Green Master Mix (Applied Biosystems) were used to perform quantitative real-time PCR and primers listed in Table 1. Relative gene expression was generated using CFX Manager 3.1 Software (Bio-Rad, Hercules, CA).

**TABLE 1:** Nucleotide sequences for DNA primers used in quantitative real-time PCR

Gene	Forward primer	Reverse primer
*GAPDH*	5′-TTAAAAGCAGCCCTGGTGAC-3′	5′-CTCTGCTCCTGTTCGAC-3′
*CFH*	5′-GTCTCCTGACCTCCCAATATG-3′	5′-TCCACCACTTCACTGTGTC-3′
*VTN*	5′-CCTTCACCGACCTCAAGAAC-3′	5′-GAAGCCGTCAGAGATATTTCG-3′

### Statistical analyses

Statistical analysis was performed using GraphPad students *t*-test and 2way ANOVA. In all figures, * indicates *P*-value < 0.05, ** indicates *P*-value < 0.01, *** indicates *P*-value < 0.001. All experiments were replicated at least in duplicate.

## Data Availability

Files are available via NCBI accession PRJNA1089547: Homo sapiens raw sequence reads.
